# Evaluating the Clinical Utility of Sulfasalazine in the Treatment of Pyoderma Gangrenosum: A Systematic Review

**DOI:** 10.1177/12034754241238713

**Published:** 2024-03-10

**Authors:** Haleh Zabihi, Delaram Shojaei, Kyle Seigel, Roudha Al-Dehneem, Vincent Piguet, David O. Croitoru

**Affiliations:** 1 Temerty Faculty of Medicine, University of Toronto, Toronto, ON, Canada; 2 Faculty of Medicine, University of British Columbia, Vancouver, BC, Canada; 3 Division of Dermatology, Department of Medicine, Women’s College Hospital, Toronto, ON, Canada; 4 Division of Dermatology, Department of Medicine, University of Toronto, Toronto, ON, Canada; 5 Division of Dermatology, University Health Network, Toronto Western Hospital, Toronto, ON, Canada

**Keywords:** pyoderma gangrenosum, neutrophilic dermatosis, sulfasalazine, DMARD, treatment, clinical dermatology

To the Editor,

Pyoderma gangrenosum (PG) is a rare neutrophilic dermatosis characterized by painful and rapidly developing ulcers predominantly affecting the lower limbs.^1^ Most cases require systemic corticosteroids or cyclosporine with the addition of a long-term immunomodulating agent.^1^ Sulfasalazine, a disease-modifying antirheumatic drug indicated for rheumatoid arthritis and ulcerative colitis, is often utilized off-label in dermatological contexts owing to its cytokine modulation and anti-inflammatory properties.^2^ Given its favourable safety profile and anecdotal efficacy in treating PG, we performed a systematic review of all study types to describe and compare clinical outcomes in PG patients treated with sulfasalazine.

We searched MEDLINE and EMBASE databases until July 6, 2023, according to a pre-registered PROSPERO protocol (CRD42023443087), which yielded 399 articles after deduplication; 232 were excluded by abstract and a full-text review was conducted on 129 studies, with 30 being included in this review.

Within the cohort of 34 cases included in our analysis, mean patient age was 41 years (SD 19.8) with a female-to-male ratio of 1.13:1. Among PG patients, 59% (20/34) reported a complete response and 9% (3/34) had partial improvement, while 32% (11/34) experienced no response (Table 1). The included cases involved patients with a PG diagnosis and any associated systemic conditions. PG diagnosis was based on clinical presentation and histopathological evidence, which included rapidly progressing ulcers with neutrophilic infiltrate and epithelial necrosis.

**Table 1.** Demographic and Clinical Features of Patients With PG Treated With Sulfasalazine.

	Clinical improvement	No response
Number of cases, n^a^	23	11
Female/male (ratio)	15/8 (1.88:1)	3/8 (1:2.67)
Mean age (SD)	44.9 (20.0)	32.6 (17.3)
PG history, n (%)		
Location		
Lower extremities	6/23 (26.1)	3/11 (27.3)
+Trunk	1/23 (4.3)	0/11 (0.0)
+Head and neck	1/23 (4.3)	1/11 (9.1)
+Trunk	3/23 (13.0)	2/11 (18.2)
+Peristomal	0/23 (0.0)	1/11 (9.1)
+Perianal, genital, and inguinal region	1/23 (4.3)	0/11 (0.0)
Trunk	5/23 (21.7)	0/11 (0.0)
Head and neck	2/23 (8.7)	1/11 (9.1)
Other^b^	4/23 (8.7)	3/11 (27.3)
Clinical variant, n (%)^c^		
Ulcerative	13/17 (76.5)	6/8 (75.0)
+Vegetative	3/17 (17.6)	0/8 (0.0)
Vegetative	1/17 (5.9)	1/8 (12.5)
Bullous	0/17 (0.0)	1/8 (12.5)
Mean number of lesions (SD)^d^	1.6 (1.3)	1.4 (0.9)
Associated systemic disease, n (%)^e^		
Crohn’s disease	7/13 (50.0)	3/6 (50.0)
Ulcerative colitis	4/13 (33.3)	2/6 (33.3)
PASH	1/13 (8.3)	0/6 (0.0)
Rheumatoid arthritis	1/13 (8.3)	0/6 (0.0)
Seronegative polyarthritis	0/13 (0.0)	1/6 (16.7)
Mean SSZ dose, g^f^ (SD)	2.8 (1.5)	1.8 (0.8)
Mean duration of treatment, wk^g^ (SD)	10.7 (8.0)	13.8 (12.7)

Abbreviations: PASH, pyoderma gangrenosum, acne, and hidradenitis suppurativa; PG, pyoderma gangrenosum; SSZ, sulfasalazine.

a Twenty of 23 cases of clinical improvement demonstrated complete remission, while 3/23 cases demonstrated a partial response to therapy.

b Other PG locations included tongue, oropharynx, breast, peristomal, and perianal, genital, and inguinal region.

c PG clinical variant was not reported in 6/23 clinical improvement cases and 3/11 no response cases.

d Number of PG lesions was not reported in 14/23 clinical improvement cases and 6/11 no response cases.

e Associated systemic disease was not reported in 10/23 clinical improvement cases and 5/11 no response cases.

f Sulfasalazine dose was not reported in 10/23 clinical improvement cases and 5/11 no response cases.

g Sulfasalazine treatment duration was not reported in 13/23 clinical improvement cases and 7/11 no response cases. One study reported keeping the patient on a maintenance dose.

The most frequent associated systemic conditions were inflammatory bowel disease (IBD) (44%; Crohn’s 26%, ulcerative colitis 18%). A smaller proportion of subjects had concurrent inflammatory arthritis (6%) and pyoderma gangrenosum with acne and hidradenitis suppurativa (PASH) syndrome (3%). Adverse events were infrequently reported, with only 2 cases reporting a generalized maculopapular skin rash and bone marrow toxicity.

Among cases that reported details of sulfasalazine therapy, the most common daily dosage was 1 g (range: 1-6 g). Mezavant formulation was mentioned in 4 studies, with only 2 detailing dosages of 2 and 4 g/day. Sulfasalazine usage duration varied considerably, averaging 11.6 weeks (range: 1-32 weeks; Supplemental Table 1). Notably, sulfasalazine was used concomitantly with another therapy in 85% of cases. Glucocorticoids were the most prevalent concurrent therapy, typically prescribed at a daily dose of 80 mg/day (range: 20-80 mg/day). Antibiotics and azathioprine (100 mg/day; range: 100-150 mg/day) were also frequently employed in combination with sulfasalazine. Treatments for PG prior to sulfasalazine initiation included antibiotics, glucocorticoids, and dapsone, of which all except 2 cases reported no benefit.

This review is limited by potential biases inherent to the included studies such as a publication bias, incomplete reporting, and confounding by indication. Although sulfasalazine was noted to be largely effective, its benefits for PG might be linked to improving a co-existing IBD. Among 11 cases reporting PG improvement in IBD patients, 55% also noted concurrent IBD improvement. In consideration of the positive outcomes reported in patients with PG treated with adjunct sulfasalazine, without apparent adverse events, we suggest that systemic sulfasalazine can be an effective option for the treatment of PG, especially when associated with IBD. This underscores the need for prospective studies to assess sulfasalazine’s clinical utility in PG treatment.

## Supplemental Material

sj-docx-1-cms-10.1177_12034754241238713 – Supplemental material for Evaluating the Clinical Utility of Sulfasalazine in the Treatment of Pyoderma Gangrenosum: A Systematic Review Supplemental material, sj-docx-1-cms-10.1177_12034754241238713 for Evaluating the Clinical Utility of Sulfasalazine in the Treatment of Pyoderma Gangrenosum: A Systematic Review by Haleh Zabihi, Delaram Shojaei, Kyle Seigel, Roudha Al-Dehneem, Vincent Piguet and David O. Croitoru in Journal of Cutaneous Medicine and Surgery
