# Supplementary material for: Evaluating the Clinical Utility of Sulfasalazine in the Treatment of Pyoderma Gangrenosum: A Systematic Review
Source: J Cutan Med Surg. 2024 Mar 10;28(3):289–91. doi: 10.1177/12034754241238713 (PMC11141088; doi:10.1177/12034754241238713)
Supplement: sj-docx-1-cms-10.1177_12034754241238713 – Supplemental material for Evaluating the Clinical Utility of Sulfasalazine in the Treatment of Pyoderma Gangrenosum: A Systematic Review [file sj-docx-1-cms-10.1177_12034754241238713.docx]

Supplemental Table 1. “Therapeutic Context of Sulfasalazine Use in Patients with PG”

|  | **Clinical improvement** | **No response** |  | **Clinical improvement** | | **No response** |
| --- | --- | --- | --- | --- | --- | --- |
| **Number of Cases, n**^a^ | 23 | 11 |  | 23 | 11 | |
| **Concomitant treatments, n (%)^b^** | | | **Previously used therapy, n (%)^e^** | | | |
| **Antibiotics** |  |  | **Antibiotics** |  |  | |
| Antibiotics alone | 1/19 (5.2) | 0/10 (0.0) | Antibiotics alone | 1/9 (11.1) | 0/7 (0.0) | |
| + Biologics | 1/19 (5.2) | 0/10 (0.0) | + Other | 1/9 (11.1)**^f^** | 2/7 (28.6)**^g^** | |
| **Steroids** | | | + Immuno-suppressant | 1/9 (11.1) | 1/7 (14.3) | |
| Steroids alone | 5/19 (26.3) | 3/10 (30.0) | **Steroids** |  |  | |
| + Immuno-suppressant | 3/19 (15.8) | 1/10 (10.0) | Steroids alone | 3/9 (33.3) | 0/7 (0.0) | |
| + Biologics | 1/19 (5.2) | 1/10 (10.0) | + Antibiotics | 1/9 (11.1) | 2/7 (28.6) | |
| + Antimicrobial | 1/19 (5.2) | 1/10 (10.0) | + Other Immunosuppressant | 1/9 (11.1) | 0/7 (0.0) | |
| + Azathioprine | 0/19 (0.0) | 1/10 (10.0) | **Biologics** | 0/9 (0.0) | 2/7 (28.6) | |
| + Other^c^ | 4/19 (26.3) | 2/10 (20.0) | **Other** | 1/9 (11.1)^h^ | 0/7 (0.0) | |
| **Other**^d^ | 3/19 (10.5) | 1/10 (10.0) |  |  |  | |

^a^20/23 cases of clinical improvement demonstrated complete remission, while 3/23 cases demonstrated a partial response to therapy.

**^b^**Concomitant treatment was not reported in 4/23 clinical improvement cases and 1/11 no response cases.

^c^Treatment included skin grafts, lamivudine, ileostomy revision, colchicine, methotrexate, and erythropoietin.

^d^Treatment included dapsone, 6-mercaptopurine, amino acids, folic acid, and vitamin C.

^e^Previously used therapy was was not reported in 14/23 clinical improvement cases and 4/11 no response cases.

^f^Treatment included high-pressure oxygen and intravenous immunoglobulin therapy.

^g^Treatment included surgical debridement, acyclovir, thalidomide, iso-tretinoin, colchicine, cyclophosphamide, split-thickness skin grafts, high-pressure oxygen, and cyclosporine.

^h^Treatment was a proctectomy.
